# Pollinator Diversity and Phenological Interplay: Exploring Mineral, Hormonal, Sugar, and Vitamin Contents in *Vitis vinifera* L. cv Bozcaada Çavuşu

**DOI:** 10.3390/plants13121612

**Published:** 2024-06-11

**Authors:** Ozkan Kaya, Hava Delavar, Fadime Ates, Muge Sahin, Nurhan Keskin, Turhan Yilmaz, Metin Turan, Harlene Hatterman-Valenti

**Affiliations:** 1 Erzincan Horticultural Research Institute, Republic of Türkiye Ministry of Agriculture and Forestry, 24060 Erzincan, Türkiye; 2 Department of Plant Sciences, North Dakota State University, Fargo, ND 58102, USA; 3 Manisa Viticulture Research Institute, Republic of Türkiye Ministry of Agriculture and Forestry, 45125 Manisa, Türkiye; 4 Department of Horticulture, Faculty of Agriculture and Natural Sciences, Bilecik Şeyh Edebali University, 11230 Bilecik, Türkiye; 5 Department of Horticulture, Faculty of Agriculture, Van Yüzüncü Yıl University, 65090 Van, Türkiye; 6 Department of Horticulture, Faculty of Agriculture, Kahramanmaraş Sütçü Imam University, 46040 Kahramanmaraş, Türkiye; 7 Faculty of Economy and Administrative Science, Yeditepe University, 34755 Istanbul, Türkiye

**Keywords:** berry phenological stages, female dominance, cultivation practices, high-quality yields

## Abstract

Unraveling the intricate physiological and biochemical intricacies associated with female dominance in grape berries across diverse developmental stages is imperative for optimizing grape production and ensuring the attainment of high-quality yields. This study conducted a thorough analysis of grape berries across phenological stages (BBCH-79, BBCH-81, BBCH-89) and cultivars. At BBCH-89, Bozcaada Çavuşu*Vasilâki demonstrated the highest berry weight and total soluble solids (TSS) levels, emphasizing its enological potential. Acidity peaked at BBCH-79 (28.16) and declined at BBCH-89 (6.11), signaling a shift towards lower acidity in later stages. Bozcaada Çavuşu*Vasilâki consistently showed the highest maturity index (MI). Mineral content variations were observed across nitrogen (N), calcium (Ca), potassium (K), phosphorus (P), magnesium (Mg), sulfur (S), iron (Fe), manganese (Mn), boron (B), zinc (Zn), and copper (Cu), with Bozcaada Çavuşu*Vasilâki often having the highest concentrations, particularly in potassium, calcium, and boron. Hormonal analysis revealed a significant surge in concentrations at BBCH-89, with Bozcaada Çavuşu*Vasilâki standing out. Notably, Indole-3-acetic acid (IAA) concentrations increased by 106%, and abscisic acid (ABA) levels peaked at BBCH-79 with a 38% increase in Bozcaada Çavuşu*Kuntra. Sugar content analysis showed variations in fructose, glucose, sucrose, rhamnose, xylose, galactose, and arabinose levels across sampling times and cultivars. Bozcaada Çavuşu*Vasilâki consistently exhibited higher sugar levels, especially at BBCH-81 and BBCH-89. Vitamin concentrations varied temporally and among cultivars, with BBCH-89 displaying the highest vitamin A concentration (6.24 mg/100 g FW), and Bozcaada Çavuşu*Vasilâki often exhibiting maximum values for vitamin B1, B2, B6, and C. Further research and targeted cultivation practices focusing on the unique attributes of Bozcaada Çavuşu*Vasilâki could enhance grape production efficiency, emphasizing its potential contribution to achieving consistently high-quality yields across various phenological stages.

## 1. Introduction

In contemporary vineyards, a strategic combination of female vines and hermaphroditic pollinators is customary, driven by the advantageous attributes of female cultivars, particularly their larger berry size compared to hermaphroditic counterparts [1,2]. Presently, contemporary female varieties and/or cultivars boast substantial berry sizes, ranging from 16 to 18 g, while hermaphroditic counterparts are confined to 10–13 g [3]. Despite the size advantage, female cultivars often contend with lower yields attributed to insufficient pollination and smaller cluster sizes [3], prompting ongoing efforts to develop new hermaphroditic cultivars featuring both substantial berry size and elevated yields [4]. Historical recommendations advised planting pollinator vines every third vine in every third row [5], a practice persisting with minimal formal adjustments in pollinator placement recommendations [6]. In contrast to the dioecious nature of wild *Vitis vinifera*, where male and female flowers occur on separate plants [7,8], the majority of cultivated grape varieties and/or cultivars possess hermaphrodite flowers with well-developed female and male organs and gametophytes [9,10,11]. These varieties and/or cultivars are inherently fit for self-pollination and successful fertilization, ensuring a “normal” proportion (>50%) of ovaries in the inflorescence that mature into seeded fruits [11,12,13]. Flowers of these cultivars, characterized by a captivating morphological androgyny, intricately balance physiological female structures with both female and male reproductive organs within the same floral architecture [8,9,10]. Additionally, rare *Vitis vinifera* varieties and/or cultivars exhibit developmental arrest in one set of reproductive organs, leading to the formation of nonfunctional organs [14]. Acknowledging *Vitis vinifera* as a predominantly self-pollinating (self-compatible) species with a mere 1–2% cross-pollination rate [15,16,17], defects in the development of flower organs or gametophytes result in diminished fertilization [18], consequently contributing to a significant proportion of undeveloped and seedless berries in select *Vitis vinifera* varieties and/or cultivars [16,19].

Bozcaada Çavuşu is a wine grape variety grown in Bozcaada, Çanakkale province of Turkey. This local variety is used in wine production in Bozcaada and makes significant contributions to the region’s wine industry. Wines produced from Bozcaada Çavuşu are available in limited quantities in Türkiye and have a regional commercial value [20,21,22]. The expression of female dominance in certain cultivars, exemplified by Bozcaada Çavuşu, is characterized by a distinctive downward curvature of the stamens, highlighting the physiological intricacies and adaptations that contribute to the overall reproductive strategy of grape varieties [20,21,22]. Bozcaada Çavuşu, despite possessing a morphologically male flower structure, exhibits physiological femaleness, necessitating the presence of pollinator varieties in vineyards due to its inability to self-pollinate [20,21,22]. Understanding the intricate floral structure becomes imperative to comprehend the significance of fertilization in grape varieties. The unique downward curvature of the stamens, though aesthetically intriguing, signifies a dependence on external agents for effective pollination [20,21,22]. Studies suggest that functionally female flowers are a rare occurrence in certain varieties, particularly those of special significance for cultivation in specific regions [8,9,10]. The morphological sterility of Bozcaada Çavuşu’s pollen, lacking fully formed openings for germination, precludes its ability to germinate, leading to fruit setting failure in self-fertilization conditions [22]. Previous research unequivocally indicates that the reproductive characteristics of Bozcaada Çavuşu, notably morphological sterility, necessitate its cultivation alongside other varieties capable of serving as pollenizers for successful fertilization and fruiting [20,21,22]. Identifying suitable pollenator varieties such as Kuntra and Vasilâki holds practical importance for establishing new vineyards. Kuntra is a table grape variety, and Vasilâki is a wine grape variety. These varieties are grown in Turkey and are of great importance for the country’s grape and wine industry, but they are not commercially cultivated. Pollination and fertilization, being fundamental factors influencing fruit setting volume, are critical considerations for fruit producers aiming to achieve high-quantity and -quality yields in the horticultural industry [20,21,22,23]. Grapes, distinguished for their delightful taste and versatile applications, hold a important role in global agriculture and culinary traditions [24,25]. The extensive utility of grapes in culinary practices, encompassing fresh consumption and the production of jams, juices, and raisins, indicates their adaptability and widespread incorporation into various cuisines [26,27]. Furthermore, grapes play a critical and non-negligible role in the wine industry, making substantial contributions to both the global economy and cultural heritage [28]. Examining the nutritional profile of grapes reveals their significance beyond taste, offering essential minerals and vitamins crucial for human health [29,30]. Specifically, grapes are rich in potassium and manganese, supporting electrolyte balance and bone health [26,27]. Additionally, they serve as a notable source of vitamin C, a potent antioxidant vital for immune function and skin health [25]. The natural sugar content in grapes, primarily in the form of fructose, provides a healthier alternative to refined sugars, imparting quick and sustained energy [25,29]. Furthermore, grapes harbor polyphenols, including resveratrol, associated with diverse health benefits such as cardiovascular protection and anti-inflammatory properties [31]. Collectively, these nutritional components position grapes as a wholesome and health-promoting fruit. On the other hand, limited studies exist on the complex interplay between floral morphology, reproductive strategies, and nutritional attributes in grape varieties, particularly focusing on the distinctive characteristics of Bozcaada Çavuşu. Understanding the floral intricacies, including the unique downward curvature of stamens in Bozcaada Çavuşu, becomes imperative for cultivating these varieties alongside suitable pollinator counterparts. This knowledge is crucial for grape producers seeking to optimize fruit setting volume, achieve high-quality yields, and contribute to the broader understanding of grapevine reproductive biology and nutritional composition. The purpose of delving into this study is to unveil the intricacies of reproductive strategies, such as the expression of female dominance and physiological femaleness in Bozcaada Çavuşu cultivar, and their impact on substances such as vitamins, hormones, minerals, and sugars.

## 2. Materials and Methods

### 2.1. Plant Material

The investigation was conducted on 30-year-old grapevine varieties, Bozcaada Çavuşu, Kuntra (Karasakız) and Vasilâki, all grafted onto 5 BB rootstock, at Bozcaada in Türkiye (located at 26°05′40″ east longitude and 39°49′11″ north latitude). The research took place in two distinct vineyards, designed to facilitate open pollination conditions for the grape varieties under study. The vineyard layout followed a 2/4 pattern trial design. Two Kuntra vines were planted alongside four Bozcaada Çavuşu vines, and similarly, two Vasilâki vines were positioned next to four Bozcaada Çavuşu vines in the other vineyard setting. The investigation involved two trials in 2022: one for pollinating Bozcaada Çavuşu grapes with Kuntra, and the other with Vasilâki. The paternity (pollinator) ratio was 2/4, meaning two Kuntra or Vasilâki vines for every four Bozcaada Çavuşu vines. The Bozcaada Çavuşu grape variety is known for its large, amber-yellow berries with a distinctive flavor, juicy content, and thin skin. The grape clusters are particularly large, winged-conical in shape, and relatively sparse in density. For pollination, the Bozcaada Çavuşu was crossed with Kuntra and Vasilâki pollinator varieties in the specific 2/4 ratios, following the method outlined by Dardeniz et al. [22]. Vasilâki, ripening mid-season, yields yellow, round, medium-large berries, and cylindrical, small-to-medium, dense clusters. Also, Kuntra is a mid-season ripening variety with reddish-purple, round, large berries, and winged-conical, large, dense clusters. The ‘Bozcaada Çavusu*Kuntra’ and Bozcaada Çavuşu*Vasilâki samples refer to the berries of the ‘Bozcaada Çavusu’ variety that have been pollinated by the ‘Kuntra’ and Vasilâki variety, and do not represent a separate variety resulting from a cross between the two. Cultural practices included regular pruning, canopy management, soil management, integrated pest management, and precise nutrient management tailored to the vineyard’s conditions. The vines were planted in a 1.4 × 1.4 m goble trellis system, with 12–15 shoots per plant after spur pruning. This spacing allowed for optimal sunlight exposure and air circulation, essential for vine health and disease prevention. The vineyard practiced dry-farming, with no irrigation employed during the year. Sample collections were randomly conducted from the upper, middle, and lower sections of the clusters, with three collection instances corresponding to BBCH-79, BBCH-81, and BBCH-89 stages, following Lorenz et al. [32] guidelines.

### 2.2. Cluster Properties and Must Composition

In our study, the berries were manually pressed using a hand press, and the obtained grape had to be immediately analyzed. The study was planned with three replications. The juice was then centrifuged for 7 min at 4000 revolutions per minute (rpm) using a Hettich Zentrifugen Universal 320 centrifuge from Germany. The resulting clarified juice was subsequently used for the evaluation of titratable acidity (TA), maturity index (MI-Brix), and total soluble solids (TSS). The MI was calculated using the formula MI = TSS*pH^2^ [24]. Total soluble solids were measured using a digital refractometer (BRX-242 Erma, Tokyo, Japan). The maturity index and TA were determined using an auto titrator. Specifically, the samples were analyzed for TA using a G20S auto titrator from Mettler Toledo, Switzerland, with 0.1 M sodium hydroxide (NaOH), and the results were expressed in g/L. The berry dimensions, including width and length, were measured using a precise caliper, while the berry weight was determined using a high-precision balance.

### 2.3. Mineral Analysis in Grape Varieties

Grape samples from the Kuntra, Bozcaada Çavuşu*Kuntra, Vasilâki, and Bozcaada Çavuşu*Vasilâki, collected at various developmental stages (as outlined in Section 2.1), were dried in an oven at 68 °C for 48 h. The study was planned with three replications. After drying, the samples were ground into a fine powder. The total nitrogen content in the samples was determined using the Kjeldahl method, employing a Vapodest Rapid Kjeldahl Distillation Unit (Gerhardt, Königswinter, Germany). The method followed was in accordance with AOAC guidelines. Macroelements (potassium, magnesium, phosphorus, sodium, and calcium) and microelements (iron, zinc, sulfur, chlorine, copper, manganese, and boron) were determined utilizing an inductively coupled plasma spectrophotometer (Optima 2100 DV, Perkin-Elmer, Shelton, CT, USA). The analytical procedure was performed following the guidelines specified by Food and Kjeldahl [33].

### 2.4. HPLC Analysis for Hormone Identification in Grape Varieties

To identify hormones in grape varieties, berry samples underwent initial homogenization and triple filtration into an 80% ethanol solution (based on 5 g of fresh weight). The study was planned with three replications. Subsequently, 200 pmol of 13C6-Indole-3-Acetic Acid (13C6-IAA) and d6-Abscisic Acid (d6-ABA) were added as internal standards. The resulting solution was concentrated using a rotary evaporator, pH-adjusted to 2.8 with dilute hydrochloric acid, and filtered through a 0.22 μm membrane filter. A partition extraction step was then conducted with diethyl ether, followed by concentration and filtration with a 0.22 μm membrane filter. Extracts underwent fractionation using an Agilent 1200 Series HPLC system with an ultraviolet detector. A Zorbax Eclipse-AAA C-18 column was used for isocratic elution with a solution of 40% ethanol and 0.1% acetic acid. Eluates, corresponding to the retention times of Indole-3-Acetic Acid (IAA) and Abscisic Acid (ABA), were separately collected. These IAA and ABA fractions were dried under reduced pressure and further purified using the same HPLC system under isocratic elution conditions. Chromatographic parameters for hormone identification and quantification followed previous reports by Kojima et al. [34]. Gibberellin (GA_3_) analysis followed the methodology by Kojima et al. [34]. In an 80% ethanol solution (based on 9 g of fresh weight), 200 pmol of d2- GA_3_ was introduced. After concentration to 20 mL, pH adjustment to 3.5 with dilute hydrochloric acid, and filtration through a 0.22 μm membrane filter, partition extraction with ethyl acetate was performed according to Kojima et al. [34]. Further extraction, separation, and purification of GA_3_ followed the described methodologies. Salicylic acid (SA) analysis was based on the method by Kaya et al. [25], with modifications. Samples were reduced to a fine powder in liquid nitrogen using a mortar and pestle, and 100 mg of the sample was combined with extraction solvents. SA was separated and quantified using an Agilent 1200 Series HPLC system with a photodiode array detector. A 5 μL sample was injected into the HPLC system, using a Zorbax Eclipse-AAA C-18 column (5 μm, 4.6 × 250 mm) at 25 °C. Mobile phases consisted of 0.3% phosphoric acid in water (solvent A) and 100% methanol (solvent B). The flow rate was set at 0.8 mL min^−1^, with the solvent system programmed through several stages, including an isocratic phase of solvent B, a gradient of solvent B, and maintenance at 100% B. Data acquisition and analysis utilized YL-clarity 4.0 software (Prague, Czech Republic), with SA content calculated using an external standard.

### 2.5. HPLC Analysis for Sugar Identification in Grape Varieties

In this study, we utilized a high-performance liquid chromatography (HPLC) method coupled with evaporative light scattering detection (ELSD) to quantitatively assess soluble sugars in the grape samples. The study was planned with three replications. The analysis was conducted using a Waters e2695 separations module equipped with an Alltech (Nicholasville, KY, USA) 3300 ELSD detector. Separation was achieved by employing a specialized XBridgeTM Amide column with dimensions of 4.6 mm inner diameter and 250 mm length, featuring 3.5 μm sized particles. Prior to analysis, both samples and standards underwent meticulous sample preparation. This involved filtering all samples and standards through 0.45 μm Millipore filters. Berries (5 g) were homogenized using an Ultra-turax homogenizer with 0.5 mL of 70% perchloric acid, followed by centrifugation at 10,000 rpm for 10 min. After the supernatant was recovered, it underwent filtration over a 0.22 mm membrane and was diluted with 10% perchloric acid to the initial homogenate concentration. The final filtered sample (0.45 μm) was then introduced into the HPLC system. Subsequently, 10 μL of each filtered sample was injected into the HPLC instrument for analysis. The HPLC-ELSD conditions were optimized following the methodology outlined in Ma et al. [35]. The mobile phase for chromatographic separation comprised a solvent mixture with 85% acetonitrile and 15% water (*v*/*v*). A consistent flow rate of 1 mL min^−1^ was maintained throughout the analysis. The column temperature and drift tube temperature were set to 45 °C and 82 °C, respectively. The nebulizer gas flow rate was maintained at 2 mL min^−^^1^. For quantification of soluble sugars in the samples, HPLC-grade sugar calibration standards (Sigma–Aldrich, Shanghai, China) were employed. The concentration of soluble sugars in the samples was determined by comparing peaks in the chromatograms with these standards (Sigma, Castle Hill, Australia). This HPLC-ELSD method, outlined for the first time by Ma et al. [35], ensures a reliable and reproducible quantification of soluble sugars in raisin samples, making it well-suited for applications in food analysis and quality control.

### 2.6. HPLC Analysis for Vitamin Identification in Grape Varieties

During the analysis, samples were initially weighed and combined with 2.5 mL of an extraction solution, varying based on the specific analysis: 8% acetic acid for 3-Methylphenylacetic acid (MPA) extraction, 0.1% oxalic acid for oxalic acid extraction, and 3% MPA. The study was planned with three replications. This mixture underwent titration with an indophenol solution (comprising 25% 6-Dichloroindophenol (DCIP) and 21% NaHCO_3_ in water) until a distinct rose-pink color appeared. For vitamin A analysis, 0.5 g samples were immersed in 20 mL of ethanol and subjected to a 30 min water bath at 85 °C. After cooling, the solution was filtered through a separator funnel. Subsequently, heptane (10 mL) was added, followed by shaking for 5 min. To facilitate layering, 20 mL of a 1.25% sodium sulfate solution was introduced into the tubes, and shaking was conducted for 2 min. Total tocopherols in the samples were determined through their reaction with cupric ions and complexation with 2,20-biquinoline (cuproine), following Samydurai et al. [36]. The solution was then poured into a conical flask, to which 25 mL of the extraction solution was added. A shaking water bath at 70 °C for 40 min was employed to sonicate the solution. After cooling, the samples were filtered with the extraction solution to reach a final volume of 50 mL. The solution for raisins underwent further filtration using 0.45 μm filter tips, and 20 μL aliquots of the solution were injected into the HPLC using an autosampler. An analytical reversed-phase C-18 column (STR ODS-M, 150 mm × 4.6 mm I.D., 5 μm, Shimadzu Corporation, Tokyo, Japan) was utilized for the separation of B complex vitamins in the berry samples. The mobile phase consisted of a mixture of 100 mM sodium phosphate buffer (pH 2.2) containing 0.8 mM sodium-1-octane sulfonate and acetonitrile at a 9:1 (*v*/*v*) ratio at 40 °C. The flow rate was maintained at a constant 0.8 mL min^−1^, and a PDA detector was employed with an absorption wavelength of 270 nm. Detection and quantification of B vitamins followed the methodology described by Mozumder et al. [37].

### 2.7. Statistical Analysis

All descriptive analyses were conducted using the Agricolae package in R Studio [38]. The influence of sampling time, cultivar, and their interactions on element, hormone, sugar, vitamin, and berry features was examined through ANOVA using the stats package in R Studio [39]. A model incorporating all main effects and interaction effects was tested for normality assumptions. Linear models were employed to assess the main effects (sampling time and cultivar) on element, hormone, sugar, vitamin, and berry features. Post hoc analysis using Tukey HSD was performed with the Agricolae package in R Studio [38]. Principal component analysis (PCA) for element, hormone, sugar and vitamin was carried out using ggbiplot within R Studio [40]. The heatmap was conducted via the package called pheatmap in R Studio [41].

## 3. Results

The results of the analysis conducted on grape berries at different phenological stages (BBCH-79, BBCH-81, and BBCH-89) and across various varieties (Kuntra, Bozcaada Çavuşu*Kuntra, Vasilâki, and Bozcaada Çavuşu*Vasilâki) are presented in Table 1. Significant differences were observed in all the examined parameters due to both the sampling time (S) and variety (C). The interaction between sampling time and variety (S x C) did not yield statistically significant results for all parameters. The berry weight, berry width, and berry length exhibited substantial variations across sampling times (S) and varieties (C). At BBCH-89, the berry weight significantly increased to 4.58 g/berry, while Bozcaada Çavuşu*Vasilâki consistently demonstrated the highest values for these parameters among cultivars. In contrast, Vasilâki exhibited the lowest values, indicating a distinct morphological pattern among cultivars at this developmental stage. TSS levels demonstrated a remarkable increase at BBCH-89 (19.30 °Brix), emphasizing the progression of sugar accumulation during grape ripening. Bozcaada Çavuşu*Vasilâki consistently exhibited the highest TSS (°Brix), aligning with its potential for enological characteristics associated with berry ripening. Acidity levels, with a peak at BBCH-79 (28.16 g/L), indicated a temporal variation across sampling times, and Bozcaada Çavuşu*Kuntra showed the highest acidity among the cultivars. The decline in acidity at BBCH-89 (6.11 g/L) suggests a shift towards lower acidity during later developmental stages. The maturity index, influenced by both sampling time and cultivar, consistently showed the highest values for Bozcaada Çavuşu*Vasilâki (31.62 MI-°Brix) across all sampling times. Table 2 presents the mineral content (mg kg^−^^1^) of grapes, including N (%), P (%), K (%), Ca, Mg, S, Mn, Fe, Zn, B, and Cu, harvested at different phenological stages (BBCH-79, BBCH-81, and BBCH-89) and across various varieties (Kuntra, Bozcaada Çavuşu*Kuntra, Vasilâki, and Bozcaada Çavuşu*Vasilâki). Significant differences were observed in the mineral content due to both the sampling time (S) and variety (C). Nitrogen levels were highest at BBCH-89, where Bozcaada Çavuşu*Vasilâki demonstrated the greatest concentration among varieties. While phosphorus levels exhibited no significant differences across sampling times, cultivar variations were evident, with Kuntra displaying the highest levels. Potassium levels, significantly influenced by both sampling time and variety, were generally higher at BBCH-79, and Bozcaada Çavuşu*Vasilâki stood out with the highest potassium content. Although calcium levels did not differ with sampling time, significant variances among cultivars were observed, and Bozcaada Çavuşu*Vasilâki exhibited the highest calcium levels. Magnesium levels, influenced by both sampling time and variety, were generally higher at BBCH-79, with Kuntra having the highest magnesium content. Sulfur levels, significantly affected by both sampling time and variety, were generally elevated at BBCH-89, and BBCH-79 displayed the highest sulfur content. Manganese, Fe, and Zn levels exhibited significant variations due to both sampling time and variety, with BBCH-89 generally presenting higher concentrations. Boron levels, significantly influenced by both sampling time and variety, were generally elevated at BBCH-89, and BBCH-79 showed the highest boron content. Copper levels, without significant differences across sampling times, varied notably among varieties, with Kuntra having the highest copper levels.

In the hormone analysis of grape berries at different phenological stages (BBCH-79, BBCH-81, and BBCH-89) across various varieties (Kuntra, Bozcaada Çavuşu*Kuntra, Vasilâki, and Bozcaada Çavuşu*Vasilâki), a detailed examination of the hormonal profiles provided valuable insights into the dynamic physiological changes [Table 3]. At BBCH-89, a significant surge in hormone concentrations was evident, with Bozcaada Çavuşu*Vasilâki standing out among varieties. The IAA concentration notably increased, showcasing a 106% elevation in Bozcaada Çavuşu*Vasilâki compared to Kuntra. ABA concentrations did not show temporal variations but revealed substantial differences among varieties. Bozcaada Çavuşu*Kuntra presented the highest ABA levels at BBCH-79, demonstrating a 38% increase compared to Kuntra. GA_3_ concentrations displayed significant variations across both sampling time and varieties. BBCH-89 generally exhibited the highest GA_3_ levels, with Bozcaada Çavuşu*Vasilâki displaying a 41% increase compared to Kuntra. SA levels did not vary temporally but showed differences among cultivars. BBCH-89 generally displayed the highest SA concentrations, with Bozcaada Çavuşu*Vasilâki exhibiting a substantial 63% increase compared to Bozcaada Çavuşu*Kuntra. Cytokinin concentrations were significantly influenced by both sampling time and variety, with Kuntra displaying a notable 44% increase compared to Bozcaada Çavuşu*Vasilâki at BBCH-89. Zeatin concentrations showed no temporal differences but varied among varieties, with Bozcaada Çavuşu*Vasilâki exhibiting a considerable 60% increase compared to Kuntra at BBCH-89. Jasmonic acid concentrations did not exhibit significant variations due to sampling time or among varieties. The sugar content (mol kg^−1^ berry) of grapes, including sucrose, glucose, fructose, rhamnose, galactose, xylose, and arabinose, was investigated at different phenological stages (BBCH-79, BBCH-81, and BBCH-89) for the varieties Kuntra, Bozcaada Çavuşu*Kuntra, Vasilâki, and Bozcaada Çavuşu*Vasilâki. Significant variations were observed across sampling times (S) and varieties (C). In terms of sampling time (S), there were substantial differences in sugar content. For instance, at BBCH-81, sucrose exhibited a significant increase from BBCH-79 (0.44 mol kg^−1^ berry) to BBCH-81 (2.28 mol kg^−1^ berry), along with significant increases in glucose and fructose. The changes were statistically significant and indicated developmental influences on sugar accumulation. Notably, BBCH-89 displayed similar trends, suggesting ongoing metabolic shifts during grape maturation. Concerning variety (C) differences, while there were no significant variations in sugar content among varieties for most sugars, BBCH-79 showed distinct patterns. The cultivar Bozcaada Çavuşu*Vasilâki consistently exhibited higher sugar levels compared to other varieties, especially at BBCH-81 and BBCH-89 [Table 4].

Table 5 presents a detailed dataset encompassing Vitamin A, Vitamin B1, Vitamin B2, Vitamin B6, and Vitamin C concentrations. Regarding Vitamin A, significant differences in Vitamin A concentrations were observed across sampling times (*p* = 0.0008). Specifically, BBCH-89 exhibited the highest concentration of Vitamin A (6.24 mg/100 g FW), while BBCH-79 and BBCH-81 recorded lower levels of 4.57 and 5.81 mg/100 g FW, respectively). Among cultivars, no statistically significant differences were observed, with values ranging from 4.77 mg/100 g FW (Kuntra variety) to 5.93 mg/100 g FW (Bozcaada Çavuşu*Vasilâki). For Vitamin B1, thiamine concentrations displayed temporal and cultivar-dependent variations. BBCH-81 recorded the highest levels (12.03 mg/100 g FW), and Bozcaada Çavuşu*Vasilâki exhibited the maximum among varieties (10.71 mg/100 g FW). Statistically significant differences between BBCH-79 and BBCH-89 underscored the impact of developmental stages on thiamine levels. Considering Vitamin B2, the investigation unveiled statistically significant differences in riboflavin concentrations across sampling times and cultivars (*p* = 0.0001). BBCH-81 displayed the highest levels (21.80 mg/100 g FW), with Bozcaada Çavuşu*Vasilâki exhibiting the maximum among cultivars (21.50 mg kg^−1^). Notably, BBCH-79 showed lower levels (10.10 mg/100 g FW). For Vitamin B6, significant variations in pyridoxine concentrations were observed across sampling times and cultivars (*p* = 0.0001). BBCH-81 exhibited the highest levels (63.70 mg/100 g FW), with Bozcaada Çavuşu*Vasilâki recording the maximum among varieties (64.90 mg/100 g FW). Contrasts between BBCH-79 and BBCH-89 were statistically significant. Given Vitamin C, the analysis highlighted significant temporal variations in ascorbic acid concentrations (*p* = 0.0001), with BBCH-81 recording the highest levels (58.40 mg/100 g FW). Bozcaada Çavuşu*Vasilâki exhibited the maximum among varieties (64.40 mg/100 g FW).

**Figure 1:**
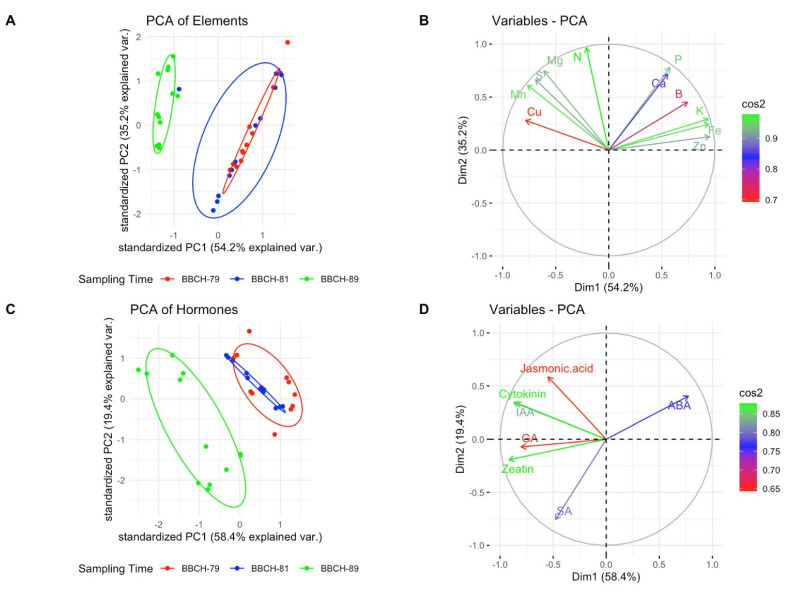

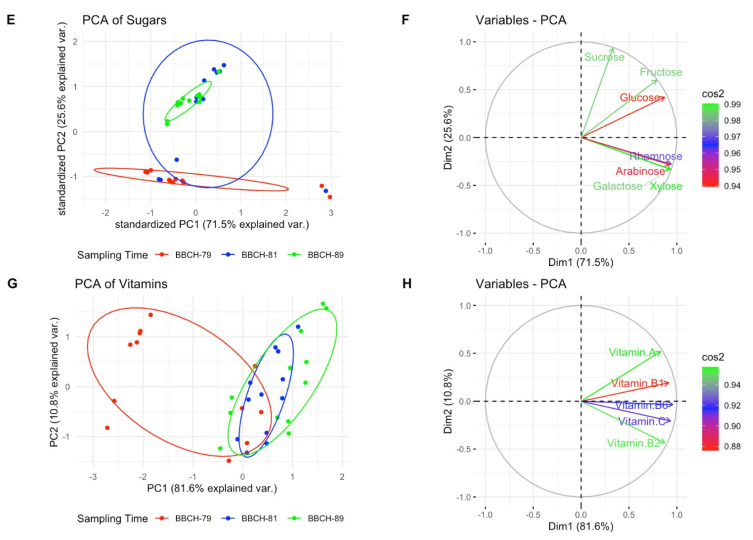
PCA biplot of berries colored by treatments. Minerals (**A**,**B**), vitamins (**C**,**D**), sugar content (**E**,**F**), and hormone content (**G**,**H**) are demonstrated.

**Figure 2:**
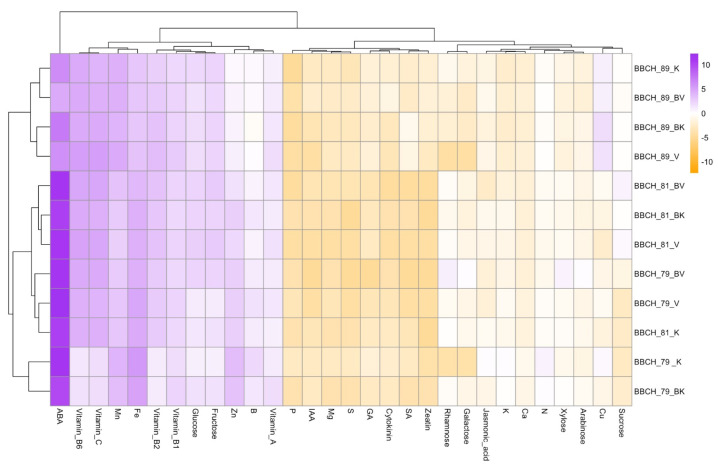
A heatmap analysis that scrutinizes numerous components. Minerals, vitamins, sugar content and hormone content are demonstrated.

Figure 1 presents PCA biplots of grape berries, categorized by phenological stage, to visually represent relationships and variances among various phytochemical components, including minerals (A), vitamins (B), sugar content (C), and hormone content (D). In the mineral biplot (Figure 1A,B), the first principal component (Dim1) accounts for 54.2% of the variance, and the second (Dim2) accounts for 35.2%. Mg, Mn, S, and Cu are closely aligned with the negative end, while Ca, B, K, Fe, and Zn align with the positive end of Dim2, indicating strong correlations. For hormones (Figure 1C,D), Dim1 explains 58.4% of the variance, and Dim2 explains 19.4%. GA, zeatin, and SA are prominently situated on the negative side, whereas ABA is on the positive side of Dim1, suggesting distinct associations. In terms of sugars (Figure 1E,F), Dim1 and Dim2 capture 71.5% and 25.6% of the variance, respectively, with sugars leaning towards the positive side of Dim1 and Dim2. Regarding vitamins (Figure 1G,H), Dim1 and Dim2 account for 81.8% and 10.8% of the variance, respectively, with vitamins closely associated with the positive side of Dim1 and Dim2. Figure 2 depicts a hierarchical clustering heatmap illustrating the relative concentrations of phytochemical components in grape samples across various cultivars and stages. Phytochemical components are clustered at the bottom of the heatmap, revealing similarities and dissimilarities between them. ABA and BBCH stages emerge as closely related, indicating similar concentration patterns across samples. Grape samples, labeled with cultivars and stages, are vertically clustered on the right. The heatmap intensity highlights that compound like Vitamin A, B6, C, B1, B2, Fe, B, Zn, Mn, glucose, fructose, and ABA have higher concentrations in specific stages, as indicated by the deep purple patches. Conversely, compounds like P, IAA, Mg, S, GA, cytokinin, SA and zeatin exhibit lower concentrations in these same samples, as shown by the deep orange color, while sucrose, Cu, arabinose, xylose, N, Ca, K, jasmonic acid, galactose and rhamnose have moderate concentrations, as demonstrated by the range of white colors. These patterns provide valuable insights into the distribution of phytochemicals in grape samples and their relationships.

## 4. Discussion

### 4.1. Effect of Sampling Time and Variety on Berry Traits and Maturity Parameters

The findings of the present study shed light on the intricate dynamics of grape berry development at various phenological stages and across different varieties, contributing to the ongoing discourse on viticulture practices. The observed variations in berry weight, width, and length across sampling times (S) and varieties (C) underscore the multifaceted nature of grape development. Morphological parameters, including berry weight, berry width, and berry length, exhibit substantial variations across sampling times and cultivars. As expected, the berries reached maximum weight and width in BBCH-89, a trend consistent with research by Keskin et al. [27], indicating a common pattern in grape weight dynamics during late phenological stages. Bozcaada Çavuşu*Vasilâki’s consistent leadership in berry weight corresponds with the cultivar-specific characteristics highlighted in the literature by Coombe and McCarthy [42], highlighting the importance of specific cultivar characteristics in determining morphological attributes. Sugar accumulation was highest in BBCH-89 (19.30), and these results were reported by Coombe, [43] and Guillaume et al. [44], highlighting the role of late phenological stages in sugar accumulation during grape ripening. Bozcaada Çavuşu*Vasilâki’s sustained high TSS (°Brix) levels align with previous research by Peña-Neira et al. [45], showcasing the cultivar’s propensity for enological attributes associated with advanced grape maturity. Temporal changes in acidity level peaked at BBCH-79 (28.16 g/L) and reached a minimum level at BBCH-89 (6.11), which is in accordance with the nature of berry development. It confirms the common pattern of acidity dynamics during berry development noted by Conde et al. [46]. Bozcaada Çavuşu*Kuntra’s elevated acidity at BBCH-79 further supports the cultivar-specific influence on acidity levels, a consistent theme in the literature. The maturity index, influenced by both sampling time and variety, consistently revealed the highest values for Bozcaada Çavuşu*Vasilâki (31.62 MI-°Brix). This aligns with the work of Guillaumie et al. [44], reinforcing the maturity index’s reliability as an indicator of grape maturity. In addition, the choice of grape pollinator individuals significantly influences berry development, as evidenced by studies such as those by Gupton [2] and Williams [1], highlighting the pivotal role of pollinators in shaping grape morphology and reproductive success. The advanced state of maturity in Bozcaada Çavuşu*Vasilâki compared to other cultivars is a recurring theme in previous research [22]. Transitioning from grape berry development to viticultural practices, the strategic combination of female vines and hermaphroditic pollinators discussed in the literature [1,2] finds relevance in our study. The challenges faced by female cultivars, such as lower yields, resonate with Conner’s observations [3], prompting ongoing efforts to develop new hermaphroditic cultivars with both substantial berry size and elevated yields [4]. Historical recommendations on pollinator placement [5] persist with minimal adjustments [6], highlighting the enduring relevance of certain viticultural practices.

### 4.2. Effect of Sampling Time and Variety on Berry Mineral Content

In the context of grape pollinator individuals, the findings suggest that the choice pollinators may contribute to variations in mineral content during different developmental stages. However, a gap in the literature exists concerning the specific impact of grape pollinator individuals on mineral composition in grapes. The mineral content analysis in Table 2 provided valuable insights into the dynamic interplay of sampling time (S) and variety (C) on grape composition. Nitrogen levels peaking at BBCH-89, with Bozcaada Çavuşu*Vasilâki displaying the highest concentration, align with studies by Ferrara et al. [47], emphasizing the temporal influence on nitrogen accumulation. This phenomenon is particularly evident at later phenological stages, such as BBCH-89, as observed in the current study. Moreover, previous studies emphasize that nitrogen accumulation is influenced by various factors, including grape varieties, soil conditions, and viticultural practices [48]. On the other hand, the absence of significant phosphorus variations across sampling times but notable cultivar differences resonate with findings in Conradie [49] highlighting the cultivar-specific nature of phosphorus uptake. While limited studies exist on the evolution of phosphorus (P) levels throughout berry development, Conradie [49] observed in Chenin Blanc grafted with 99R that P levels in the bunches consistently rise during development, culminating in levels of 641 mg per vine at the time of harvest. In our study, K+ levels, significantly influenced by both sampling time and variety, particularly higher at BBCH-79, reinforce the observations of Zhenming et al. [50], illustrating the intricate dynamics of K+ accumulation during berry growth. Authors also reported that the transportation of K+ from leaves to fruits exhibited variability depending on the stage of berry development, with more pronounced transport observed in the later stages, approximately 20 days before harvest. This underscores the significance of applying K+ in a timely and sufficient manner, both during full bloom and fruit coloring stages, to enhance fruit growth and optimize the efficiency of K+ fertilizer utilization. Variances in Ca^2+^ levels among cultivars at BBCH-89 echo the work of Conradie [49], suggesting a cultivar-specific influence on calcium uptake. Contrary to our findings, Cabanne and Donèche [51] reported that grape berries from various cultivars, including Sauvignon Blanc, Semillon, Merlot, and Cabernet Sauvignon, displayed an increase in Ca^2+^ content in the pericarp until *véraison*, followed by a decline, thereafter, indicating reduced xylem flow post-*véraison*. A similar trend was observed in the flesh, where Ca^2+^ levels peaked at approximately 0.1 mg per berry before *véraison* and gradually decreased afterward. In contrast, our study revealed that the skin and seeds exhibited a continual rise in Ca^2+^ concentrations throughout ripening, reaching a plateau around maturity and accumulating concentrations slightly surpassing those detected in the flesh. The higher magnesium content in Kuntra variety at BBCH-79 correlates with Conradie [49], emphasizing both the temporal and cultivar-related variations in magnesium levels. It is noteworthy that grape clusters played a relatively minor role in this accumulation, contributing only 15.4% to the overall Mg^2+^ content, whereas the leaves constituted the primary reservoir of this essential macronutrient. This uptake pattern aligns with the recognized structural function of Mg^2+^ in chlorophyll molecules. In contrast, existing studies propose a dual-phase accumulation of Mg^2+^ in grape berries, occurring both before and after *véraison*. The substantial post-*véraison* accumulation is in accordance with the translocation of Mg^2+^ through the phloem into the grape berry. Schaller [52] provides insights into this dynamic process, noting an initial surge in uptake rates during the early growth phase, followed by a decline at *véraison*, and culminating in a secondary peak just before harvest. The elevated levels of S, Cu, and B at BBCH-89 in our results indicate the importance of considering sampling time in the accumulation of sulfur and boron. However, it is important to note that the lack of specific studies on sulfur makes it challenging to delve into detailed discussions on this aspect. However, the higher concentrations of Mg^2+^, iron, and zinc at BBCH-89 are consistent with the broader literature [48,49], indicating increased concentrations of these minerals in later developmental stages. In the context of grape pollinator individuals, the findings suggest that the choice of pollinator may contribute to variations in mineral content during different developmental stages. However, a gap in the literature exists concerning the specific impact of grape pollinator individuals on mineral composition in grapes. Future research focusing on the interaction between grape pollination, mineral uptake, and developmental stages would provide a more comprehensive understanding of these intricate relationships.

### 4.3. Effect of Sampling Time and Variety on Berry Hormonal Content

The hormonal analysis conducted on grape berries at distinct phenological stages (BBCH-79, BBCH-81, and BBCH-89) and across diverse varieties (Kuntra, Bozcaada Çavuşu*Kuntra, Vasilâki, and Bozcaada Çavuşu*Vasilâki) has unveiled intricate physiological changes, contributing to a nuanced comprehension of hormonal profiles [Table 3]. The substantial surge in IAA concentrations, particularly notable at BBCH-89, with Bozcaada Çavuşu*Vasilâki demonstrating a remarkable 106% elevation compared to Kuntra variety, aligns with established knowledge regarding auxin dynamics in berry development [53,54,55,56]. It is noteworthy that a significant proportion of IAA in plants exists in conjugated forms, either ester-linked to sugars or amide-linked to amino acids, peptides, and proteins. These conjugates, traditionally deemed biologically inactive, play pivotal roles in storage, transport, and IAA degradation, thereby contributing to the regulation of auxin homeostasis [55,56]. In the context of berry development, the accumulation of IAA-amide conjugates during and after the onset of ripening has been documented in various fruits, encompassing climacteric fruits such as bananas and muskmelons, as well as non-climacteric strawberries [53,54]. The formation of these conjugates, often coupled with low levels of free auxins, provides a plausible rationale for the observed diminished concentration of free auxins in ripening grape berries. This interpretation, which is consistent with our findings, is further supported by a recent investigation into Cabernet Sauvignon berries, which reported a ripening-associated accumulation of the IAA-amino acid conjugate IAA-aspartic acid (IAA-Asp). The concentration of IAA remained low in berries until the stage of *véraison*, subsequently peaking at BBCH-89. This synthesis elucidates the complex hormonal dynamics during grape berry development, enriching our understanding of the underlying regulatory processes influencing auxin dynamics. On the other hand, the observed substantial differences in ABA concentrations among grapevine varieties in our study, with Bozcaada Çavuşu*Kuntra displaying the highest levels at BBCH-79 and a 38% increase compared to Kuntra, align with the existing literature on the complexity of ABA metabolism and its regulation during berry development. Previous studies suggest that ABA exists not only in its free form but also in conjugated (bound) forms, with ABA glycosyl ester (ABA-GE) being a prevalent conjugated form [57]. The existing knowledge on ABA-GE in grapes indicates that its levels can vary during berry development, potentially acting as a storage form from which free ABA is released by hydrolysis [58]. However, the current study did not directly address the presence or role of ABA-GE in the observed ABA concentrations among cultivars at BBCH-79. The sequestration of ABA through conjugation may play a role in the decrease in free ABA observed midway through the ripening phase, as suggested by Wheeler et al. [59]. They showed that an increase in conjugated ABA accounted for approximately one-third of the observed decrease in free ABA levels, while the rest was attributed to factors such as dilution due to berry size increase, enzymic catabolism, or export from the berry. In our study, the substantial differences in ABA concentrations among cultivars at BBCH-79 may be influenced by a combination of factors, including genetic differences in ABA metabolism, responses to environmental stimuli, and possibly variations in the sequestration of ABA through conjugation.

The significant variations in GA_3_ concentrations across sampling times and varieties in our study, particularly the peak levels at BBCH-89 in Bozcaada Çavuşu*Vasilâki, resonate with the dynamic temporal changes in GA levels during grape berry development as elucidated by Teszlák et al. [60]. The congruence between our findings and the established literature indicates the pivotal role GAs play in modulating the physiological processes associated with grape berry development. The documented 41% increase in GA concentrations in Bozcaada Çavuşu*Vasilâki compared to Kuntra aligns with the cultivar-specific responses to gibberellic acid highlighted in studies by Rodriguez et al. [61]. This concordance underscores the nuanced nature of GA regulation and its interplay with the unique characteristics of different grapevine cultivars. The observed variations in GA concentrations in our study parallel the intricate effects of GA treatments on berry size and ripening reported in the literature, particularly the proposition by Teszlák et al. [60] that GA-induced berry size increase may lead to a delay in ripening. This resonates with our findings, offering a potential explanation for the observed differences among varieties at BBCH-89. Further insights are provided by the literature on the influence of GA application on invertase activity and hexose accumulation. The reported increase in invertase activity and hexose accumulation in response to GA_3_ treatments in Sultana flowers/berries [62] provides a mechanistic perspective on how GAs may impact berry development. The consistent patterns observed in our study regarding GA concentrations align with previous research, demonstrating the complex and cultivar-specific effects of GAs on grape berry development. The observed differences in SA levels among varieties, particularly the substantial 63% increase in Bozcaada Çavuşu*Vasilâki compared to Bozcaada Çavuşu*Kuntra at BBCH-89, indicate cultivar-specific variations in SA concentrations. This finding aligns with the documented role of SA in signaling defense and stress responses in plants [63]. However, the absence of temporal variations suggests a stable presence of SA throughout grape berry development, and without reported developmental roles for salicylates at this stage [63], the specific implications of the observed differences remain elusive. The role of SA is less understood, but its injection into berries has been demonstrated to delay skin color development. This evaluation synthesizes the findings with the hormones in grapes and contextualizes them within the broader literature on grape berry development [64]. In contrast, cytokinin concentrations exhibited significant influences from both sampling time and variety, with Kuntra displaying a notable 44% increase compared to Bozcaada Çavuşu*Vasilâki at BBCH-89. This aligns with the documented involvement of cytokinins in diverse plant processes [65]. The decline in cytokinin levels from one-week-old berry flesh to *véraison* is consistent with proposed roles in flower development, fruit set, and the ability of cytokinin to delay berry development [66]. The observed variations in zeatin concentrations among varieties, with Bozcaada Çavuşu*Vasilâki exhibiting a considerable 60% increase compared to Kuntra at BBCH-89, further support the notion of cultivar-specific responses to zeatin. The decline in zeatin levels in berry flesh by *véraison* aligns with proposed roles in flower development and fruit set [66]. JA concentrations did not exhibit significant variations due to sampling time or among varieties. The documented elevated levels of JA and methyl-jasmonate (MeJA) in seeded berries at 20 days after flowering, coincident with early stages of cell division and expansion, suggest potential roles in developmental processes and responses to stress [67]. However, the absence of a *véraison*-associated increase in jasmonate levels indicates no direct involvement in ripening. The information regarding cytokinin oxidase, a putative cytokinin catabolic enzyme, provides molecular insights into cytokinin metabolism during berry development. The observed high transcript levels early in berry development, decreasing to low levels approaching *véraison*, suggest a potential role in the decrease in cytokinin levels during this critical phase. This finding complements the broader understanding of cytokinin dynamics in berry development physiology.

### 4.4. Effect of Sampling Time and Variety on Berry Sugar Content

Our findings showed that sampling time (S) has a significant impact on sugar composition. Specifically, sucrose, glucose, and fructose levels were significantly higher at sampling times BBCH-81 and BBCH-89 compared to BBCH-79. This indicated the accumulation of sugar in berries during the ripening process. However, no statistically significant effect of the variety (C) factor on sugar composition was observed. This showed that sugar profiles were similar among the varieties studied. However, sucrose, glucose, and fructose levels of the Bozcaada Çavuşu x Vasilâki variety were relatively higher than those of other varieties. For other sugars such as rhamnose, galactose, xylose and arabinose, there was no significant difference between sampling time or variety. Levels of these sugars varied independently of the factors examined. The observed variations in sugar content across different phenological stages (BBCH-79, BBCH-81, and BBCH-89) and varieties (Kuntra, Bozcaada Çavuşu*Kuntra, Vasilâki, and Bozcaada Çavuşu*Vasilâki) provide valuable insights into the complex dynamics of grape sugar metabolism. The significant increase in sucrose from BBCH-79 to BBCH-81, along with parallel rises in glucose and fructose, indicated the dynamic nature of sugar accumulation during berry development. These findings align with previous studies highlighting the impact of phenological stages on sugar composition in berries [68,69]. The substantial differences observed in sugar content at BBCH-81 suggest a critical period of sugar accumulation, likely associated with the onset of *véraison* and the transition from berry growth to ripening [69]. The consistently higher sugar levels in the variety Bozcaada Çavuşu*Vasilâki at BBCH-81 and BBCH-89 are intriguing and merit attention. Genetic variability in sugar metabolism among grape cultivars is well-documented [70], and these results corroborate the notion that specific cultivars may exhibit distinct sugar accumulation patterns. The genetic regulation of sugar transporters, enzymes, and regulatory factors involved in sugar metabolism contributes to such variations [71].

### 4.5. Effect of Sampling Time and Variety on Berry Vitamin Content

The results indicate significant variations in the concentrations of several vitamins (A, B1, B2, B6, C) across different developmental stages (BBCH-79, BBCH-81, BBCH-89) and among grape varieties. These findings provide insights into the dynamic changes in the nutritional composition of grape berries during their developmental stages. In terms of Vitamin A concentrations, BBCH-89 exhibited the highest levels, emphasizing the influence of developmental stages on vitamin content. While no statistically significant differences were observed among varieties, the variations at different stages indicate the importance of considering the timing of sampling. This aligns with the broader understanding of the impact of developmental stages on nutrient accumulation in fruits [72,73]. For Vitamin B1 (thiamine), both temporal and cultivar-dependent variations were observed. BBCH-81 recorded the highest levels, and Bozcaada Çavuşu*Vasilâki exhibited the maximum among varieties. The statistically significant differences between BBCH-79 and BBCH-89 indicate the importance of developmental stages in influencing thiamine levels. This aligns with previous research highlighting the impact of developmental stages on vitamin content in fruits [73]. Significant variations in Vitamin B2 (riboflavin) concentrations were evident across sampling times and varieties. BBCH-81 displayed the highest levels, with Bozcaada Çavuşu*Vasilâki exhibiting the maximum among varieties. The lower levels at BBCH-79 emphasize the temporal dynamics of vitamin content during berry development. Such variations are consistent with previous studies on the impact of developmental stages on vitamin concentrations in fruits [73]. For Vitamin B6 (pyridoxine), the observed significant variations across sampling times and cultivars highlight the dynamic nature of pyridoxine concentrations during berry development. BBCH-81 exhibited the highest levels, and Bozcaada Çavuşu*Vasilâki recorded the maximum among varieties. The contrasts between BBCH-79 and BBCH-89 further emphasize the impact of developmental stages on pyridoxine levels. This aligns with the understanding of the temporal variations in vitamin content during fruit development [72,73]. In the case of Vitamin C (ascorbic acid), the analysis revealed significant temporal variations, with BBCH-81 recording the highest levels. Bozcaada Çavuşu*Vasilâki exhibited the maximum among varieties. The documented interest in ascorbate metabolism and strategies for increasing vitamin C concentrations in various fruits are well-founded, although grape berries are not among those with high vitamin C content [74]. Regarding tocochromanols, particularly tocopherols and tocotrienols, these molecules with vitamin E activity contribute to preventing lipid oxidation. The accumulation of α-tocopherol in grape berries, with decreasing concentrations as ripening progresses, aligns with previous research [75].

On the other hand, the results from the PCA biplots and hierarchical clustering heatmap provide a comprehensive overview of the relationships and variances among phytochemical components in grape berries, offering valuable insights into the intricate interplay of minerals, vitamins, sugars, and hormones. The PCA biplot for minerals highlights the significant roles of Mg, Mn, S, Cu, Ca, B, K, Fe, and Zn in shaping the variance among grape cultivars. The clustering of these minerals on opposite ends of Dim2 suggests distinct patterns of accumulation. The specific alignment of minerals in the biplot provides a visual representation of their correlations, aiding in understanding the complex relationships among them. The hormonal biplot illustrates the diverse roles of GA, zeatin, ABA, and SA in berry development. The positioning of ABA on the positive side of Dim1 suggests a potential association with specific stages or cultivars. This aligns with research indicating the pivotal role of ABA in berry ripening and stress responses [76]. The negative alignment of GA, zeatin, and SA suggests potential antagonistic relationships or shared regulatory pathways. The sugar biplot reveals the coordinated accumulation of sucrose, glucose, fructose, rhamnose, galactose, xylose, and arabinose during grape development. The distinct separation along Dim1 and Dim2 underscores the temporal and cultivar-dependent variations in sugar content. These findings support existing knowledge on the impact of phenological stages on sugar metabolism in grape berries [70]. The visual representation provided by the biplot enhances our understanding of the specific sugars driving the observed variances. In the vitamin biplot, the close association of vitamins with the positive side of Dim1 and Dim2 emphasizes their collective contribution to grape berry composition. This aligns with the recognized antioxidant properties of vitamins A, B6, C, B1, and B2 in grapes [77]. The biplot provides a clear visualization of the variance in vitamin composition across different varieties, contributing to a more nuanced understanding of their distribution. Regarding the hierarchical clustering heatmap, the heatmap reinforces the patterns observed in the PCA biplots, emphasizing the relative concentrations of phytochemicals in grape samples. The clustering of ABA and BBCH stages indicates synchronized concentration patterns, supporting the idea that ABA plays a regulatory role during specific developmental stages [78]. The heatmap’s intensity reveals compounds with higher concentrations, such as vitamins and certain minerals, providing a detailed spatial representation of their distribution. Once we considered heatmap analysis, which visualizes the similarity or difference between samples, along with PCA variables used to represent multidimensional datasets in fewer dimensions, our findings aligned with previous research results [79,80].

## 5. Conclusions

This comprehensive analysis of grape berries across various berry development stages and cultivars has unveiled key insights into the factors shaping grape development. Bozcaada Çavuşu*Vasilâki emerged as a standout variety, consistently exhibiting superior characteristics, highest berry weight, TSS levels, and maturity index, underscoring its enological potential. The study emphasized the significance of accounting for berry developmental stages, notably showcasing temporal variations in acidity levels and a shift towards lower acidity in later stages. Mineral content analysis highlighted distinctive patterns, with Bozcaada Çavuşu*Vasilâki often displaying elevated concentrations in key minerals like K, Ca, and B. Hormonal analysis at BBCH-89 revealed a notable surge, particularly in IAA concentrations, with Bozcaada Çavuşu*Vasilâki standing out. Sugar content analysis showcased substantial variations, and Bozcaada Çavuşu*Vasilâki consistently demonstrated higher sugar levels. Temporal and variety-based variations were evident in vitamin concentrations, with BBCH-89 displaying the highest Vitamin A concentration. Bozcaada Çavuşu*Vasilâki consistently exhibited maximum values for B1, B2, B6, and C. These findings highlight the intricate interplay of physiological and biochemical factors during berry development, emphasizing the importance of considering both developmental stages and variety selection for optimizing grape production. The study’s insights have practical implications for grape cultivation, offering valuable knowledge that can enhance farming practices and contribute to advancements in viticulture.

## Figures and Tables

**Table 1:** Berry weight, berry width and length, total soluble solid, titratable acidity and maturity index of grapes (Kuntra, Bozcaada Çavuşu*Karakiz, Vasilâki and Bozcaada Çavuşu*Vasilâki) harvested in BBCH-79, BBCH-81 and BBCH-89 phenological stages.

Sampling Time (S) ^x^	Berry Weight (g/Berry)	Berry Width (mm)	Berry Length (mm)	TSS (°Brix)	TA (g/L Tartaric Acid)	Maturity Index (MI-°Brix)
BBCH_79	1.17 ± 0.40 c	4.40 ± 0.65 c	4.60 ± 0.81 c	4.30 ± 0.38 c	28.16 ± 1.19 a	1.52 ± 0.07 c
BBCH_81	3.23 ± 0.02 b	13.17 ± 1.76 b	14.02 ± 2.27 b	11.25 ± 0.13 b	18.89 ± 1.11 b	5.98 ± 0.36 b
BBCH_89	4.58 ± 0.46 a	18.62 ± 2.13 a	19.91 ± 2.26 a	19.30 ± 1.84 a	6.11 ± 0.62 c	31.62 ± 1.74 a
Cultivar (C) ^y^						
Kuntra	2.66 ± 0.34 b	11.96 ± 2.91 c	12.24 ± 1.04 c	12.75 ± 1.59 a	18.73 ± 2.74 a	13.06 ± 1.22 b
Bozcaada Çavuşu*Kuntra	3.84 ± 0.78 a	13.79 ± 2.88 a	15.07 ± 1.59 a	10.82 ± 0.93 d	16.59 ± 2.91 d	12.60 ± 1.23 c
Vasilâki	1.67 ± 0.86 c	9.87 ± 1.29 d	9.90 ± 0.25 c	11.60 ± 1.43 b	18.39 ± 3.82 b	12.49 ± 1.52 c
Bozcaada Çavuşu*Vasilâki	3.79 ± 0.96 a	12.65 ± 2.77 b	14.15 ± 1.88 b	11.30 ± 1.10 c	17.17 ± 1.86 c	14.00 ± 1.29 a
Significance						
S	0.0001 ***	0.0001 ***	0.0001 ***	0.0001 ***	0.0001 ***	0.0001 ***
C	0.0001 ***	0.0001 ***	0.0001 ***	0.0001 ***	0.0001 ***	0.0001 ***
S x C	0.9714	0.8727	0.7865	09243	0.9875	0.9658

^x^, Mean separation in sampling time; ^y^, Mean separation in variety; S, Sampling time; C, Variety; S x C, interactions; For a given factor, different letters within a column represent significant differences (Tukey test, ***, Significant at *p*-value < 0.001). Data are stated as averages of the data and their standard errors.

**Table 2:** Minerals (mg kg^−1^) of grapes (Kuntra, Bozcaada Çavuşu*Kuntra, Vasilâki and Bozcaada Çavuşu*Vasilâki) harvested in BBCH-79, BBCH-81 and BBCH-89 phenological stages.

Sampling Time (S) ^x^	N (%)	P (%)	K(%)	Ca	Mg	S	Mn	Fe	Zn	B	Cu
BBCH_79	1.71 ± 0.12 b	0.18 ± 0.00	1.38 ± 0.06 a	0.79 ± 0.06	0.17 ± 0.02 b	0.12 ± 0.03 b	18.80 ± 2.24 b	55.8 ± 3.48 a	14.54 ± 3.41 a	5.34 ± 0.69 a	1.22 ± 0.41 b
BBCH_81	1.64 ± 0.10 b	0.16 ± 0.00	1.24 ± 0.05 a	0.75 ± 0.05	0.18 ± 0.03 b	0.13 ± 0.01 b	19.60 ± 2.64 b	49.9 ± 2.58 a	11.85 ± 5.42 a	4.77 ± 0.03 a	0.99 ± 0.39 b
BBCH_89	2.25 ± 0.15 a	0.15 ± 0.00	0.60 ± 0.05 b	0.65 ± 0.04	0.38 ± 0.02 a	0.35 ± 0.02 a	51.90 ± 2.37 a	23.3 ± 2.69 b	3.95 ± 0.52 b	2.92 ± 0.16 b	6.40 ± 0.45 a
Cultivar (C) ^y^											
Kuntra	2.25 ± 0.13 a	0.20 ± 0.00 a	1.39 ± 0.06 a	0.88 ± 0.07	0.28 ± 0.02	0.20 ± 0.02 ab	33.70 ± 2.33	55.80 ± 3.18 a	12.78 ± 6.88 a	5.42 ± 0.69	2.67 ± 0.36
Bozcaada Çavuşu*Kuntra	1.76 ± 0.14 ab	0.16 ± 0.00 ab	1.06 ± 0.04 b	0.70 ± 0.06	0.20 ± 0.03	0.18 ± 0.01 b	27.00 ± 2.88	41.80 ± 2.96 b	10.78 ± 6.09 ab	4.04 ± 0.19	3.40 ± 0.85
Vasilâki	1.61 ± 0.17 b	0.15 ± 0.00 b	0.92 ± 0.07 b	0.65 ± 0.05	0.21 ± 0.02	0.17 ± 0.03 b	26.60 ± 2.43	37.90 ± 2.76 b	8.22 ± 3.91 b	3.60 ± 0.94	2.67 ± 0.60
Bozcaada Çavuşu*Vasilâki	1.87 ± 0.10 ab	0.14 ± 0.00 b	0.92 ± 0.05 b	0.69 ± 0.04	0.28 ± 0.04	0.26 ± 0.01 a	33.10 ± 3.74	36.60 ± 3.96 b	8.66 ± 5.75 b	4.30 ± 0.67	2.72 ± 0.74
Significance											
S	0.0031 **	0.1111	0.0001 ***	0.3094	0.0001 ***	0.0001 ***	0.0001 ***	0.0001 ***	0.0001 ***	0.0007 ***	0.0001 ***
C	0.0270 *	0.0084 **	0.0003 ***	0.1323	0.1134	0.0145 *	0.1295	0.0001 ***	0.0061 **	0.0628	0.6866
S x C	0.7054	0.6957	0.6238	0.5519	0.8765	0. 7858	0.7565	0.5998	0.8958	0.7699	0.8166

^x^, Mean separation in sampling time; ^y^, Mean separation in variety; S, Sampling time; C, Variety; S x C, interactions; For a given factor, different letters within a column represent significant differences (Tukey test, *, Significant at *p*-value < 0.05; **, Significant at *p*-value < 0.01; ***, Significant at *p*-value < 0.001). Data are stated as averages of the data and their standard errors.

**Table 3:** Hormone content (ng mg^−1^) of grapes (Kuntra, Bozcaada Çavuşu*Kuntra, Vasilâki and Bozcaada Çavuşu*Vasilâki) harvested in BBCH-79, BBCH-81 and BBCH-89 phenological stages.

Sampling Time (S) ^x^	IAA	ABA	GA_3_	SA	Cytokinin	Zeatin	Jasmonic Acid
BBCH_79	0.16 ± 0.01 b	5842.60 ± 42.74 a	0.21 ± 0.06 b	0.14 ± 0.08 b	0.22 ± 0.01 b	0.04 ± 0.04 b	0.96 ± 0.12
BBCH_81	0.22 ± 0.02 b	4773.06 ± 34.54 b	0.32 ± 0.09 b	0.20 ± 0.09 b	0.20 ± 0.05 b	0.09 ± 0.03 b	1.19 ± 0.25
BBCH_89	0.33 ± 0.02 a	208.33 ± 26.31 c	0.67 ± 0.07 a	0.81 ± 0.08 a	0.58 ± 0.04 a	0.64 ± 0.07 a	1.17 ± 0.14
Cultivar (C) ^y^							
Kuntra	0.33 ± 0.02 a	2987.44 ± 67.76 c	0.53 ± 0.08	0.39 ± 0.04	0.46 ± 0.04 a	0.32 ± 0.03 a	1.61 ± 0.15 a
Bozcaada Çavuşu*Kuntra	0.21 ± 0.03 bc	1880.03 ± 38.41 d	0.33 ± 0.04	0.55 ± 0.09	0.23 ± 0.06 b	0.24 ± 0.07 ab	0.99 ± 0.13 b
Vasilâki	0.11 ± 0.05 c	5025.09 ± 42.95 a	0.36 ± 0.03	0.36 ± 0.06	0.18 ± 0.07 b	0.14 ± 0.03 b	0.84 ± 0.11 b
Bozcaada Çavuşu*Vasilâki	0.30 ± 0.04 ab	4539.41 ± 34.68 b	0.38 ± 0.04	0.24 ± 0.09	0.47 ± 0.05 a	0.35 ± 0.05 a	0.98 ± 0.18 b
Significance							
S	0.0002 ***	0.0002 ***	0.0002 ***	0.0003 ***	0.0001 ***	0.0002 ***	0.3725
C	0.0007 ***	0.0002 ***	0.3212	0.0682	0.0003 ***	0.0001 ***	0.0041 *
S x C	0.9795	0.6983	0.7741	0.8565	0.8755	0.9875	0.8232

^x^, Mean separation in sampling time; ^y^, Mean separation in variety; S, Sampling time; C, Variety; S x C, interactions; For a given factor, different letters within a column represent significant differences (Tukey test, *, Significant at *p*-value < 0.05; ***, Significant at *p*-value < 0.001). Data are stated as averages of the data and their standard errors.

**Table 4:** Sugar content (mol kg^−1^ berry) of grapes (Kuntra, Bozcaada Çavuşu*Kuntra, Vasilâki and Bozcaada Çavuşu*Vasilâki) harvested in BBCH-79, BBCH-81 and BBCH-89 phenological stages.

Sampling Time (S) ^x^	Sucrose	Glucose	Fructose	Rhamnose	Galactose	Xylose	Arabinose
BBCH_79	0.44 ± 0.24 b	6.04 ± 1.59 b	6.56 ± 1.09 b	1.82 ± 0.19	1.20 ± 0.40	1.92 ± 0.80	1.33 ± 0.23
BBCH_81	2.28 ± 1.26 a	11.16 ± 1.37 a	13.05 ± 1.24 a	1.81 ± 0.34	1.12 ± 0.97	1.63 ± 0.23	1.17 ± 0.92
BBCH_89	2.42 ± 0.21 a	9.45 ± 1.04 ab	12.48 ± 1.52 a	0.75 ± 0.70	0.47 ± 0.37	1.05 ± 0.26	0.98 ± 0.18
Cultivar (C) ^y^							
Kuntra	1.21 ± 0.33	7.50 ± 1.97	9.02 ± 1.36	1.39 ± 0.83	0.93 ± 0.27	1.52 ± 0.50	1.12 ± 0.12
Bozcaada Çavuşu*Kuntra	1.79 ± 0.53	8.76 ± 1.11	9.78 ± 1.92	1.08 ± 0.77	0.67 ± 0.42	1.24 ± 0.33	0.98 ± 0.24
Vasilâki	1.65 ± 0.15	7.69 ± 1.41	9.85 ± 1.86	0.99 ± 0.69	0.65 ± 0.41	1.10 ± 0.31	0.98 ± 0.16
Bozcaada Çavuşu*Vasilâki	2.21 ± 0.08	11.60 ± 1.36	14.13 ± 1.59	2.39 ± 0.25	1.47 ± 0.48	2.28 ± 0.96	1.56 ± 0.34
Significance							
S	0.0001 ***	0.0068 **	0.0039 **	0.0715	0.0796	0.1519	0.5495
C	0.0579	0.0881	0.1137	0.0903	0.1475	0.1205	0.3551
S x C	0.9848	0.9598	0. 9287	0.9763	0.7658	0.9449	0.8762

^x^, Mean separation in sampling time; ^y^, Mean separation in variety; S, Sampling time; C, Variety; S x C, interactions; For a given factor, different letters within a column represent significant differences (Tukey test, **, Significant at *p*-value < 0.01; ***, Significant at *p*-value < 0.001). Data are stated as averages of the data and their standard errors.

**Table 5:** Vitamins (mg/100 g FW) of grapes (Kuntra, Bozcaada Çavuşu*Kuntra, Vasilâki and Bozcaada Çavuşu*Vasilâki) harvested in BBCH-79, BBCH-81 and BBCH-89 phenological stages.

Sampling Time (S) ^x^	Vitamin A	Vitamin B1	Vitamin B2	Vitamin B6	Vitamin C
BBCH_79	4.57 ± 0.96 b	8.19 ± 0.46 b	10.10 ± 1.94 b	26.80 ± 2.13 b	27.30 ± 2.02 b
BBCH_81	5.81 ± 0.84 a	12.03 ± 0.47 a	21.80 ± 1.34 a	63.70 ± 3.97 a	58.40 ± 3.10 a
BBCH_89	6.24 ± 0.20 a	13.24 ± 0.67 a	23.00 ± 1.53 a	68.10 ± 2.14 a	62.00 ± 3.36 a
Cultivar (C) ^y^					
Kuntra	4.77 ± 0.36	9.88 ± 0.10	14.40 ± 1.69 b	43.00 ± 3.23 c	38.00 ± 2.56 b
Bozcaada Çavuşu*Kuntra	5.56 ± 0.95	10.71 ± 0.32	17.30 ± 1.76 ab	43.90 ± 3.19 bc	39.60 ± 2.74 b
Vasilâki	5.89 ± 0.43	11.58 ± 0.96	20.00 ± 1.10 ab	59.60 ± 3.83 ab	55.00 ± 3.95 a
Bozcaada Çavuşu*Vasilâki	5.93 ± 0.82	12.45 ± 0.47	21.50 ± 1.14 a	64.90 ± 4.27 a	64.40 ± 3.98 a
Significance					
S	0.0008 ***	0.0001 ***	0.0001 ***	0.0001 ***	0.0001 ***
C	0.0603	0.0635	0.0181 *	0.0011 **	0.0001 ***
S x C	0.6257	0. 5907	0.9876	0.9218	0.8748

^x^, Mean separation in sampling time; ^y^, Mean separation in variety; S, Sampling time; C, Variety; S x C, interactions; For a given factor, different letters within a column represent significant differences (Tukey test, *, Significant at *p*-value < 0.05; **, Significant at *p*-value < 0.01; ***, Significant at *p*-value < 0.001). Data are stated as averages of the data and their standard errors.

## Data Availability

The data used in this study is openly available, and the data used are available upon request from the corresponding authors O.K. and F.A.
